# Abnormal urodynamic changes in post-upper urinary tract dysfunction in ureteral obstruction rat models

**DOI:** 10.3389/fphys.2024.1341220

**Published:** 2024-01-29

**Authors:** Xin Liu, Xing Li, Limin Liao

**Affiliations:** ^1^ Shandong University, Jinan, Shandong, China; ^2^ Department of Urology, China Rehabilitation Research Center, Beijing Bo’ai Hospital, Beijing, China; ^3^ University of Health and Rehabilitation Sciences, Qingdao, Shandong, China; ^4^ China Rehabilitation Science Institute, Beijing, China; ^5^ Beijing Key Laboratory of Neural Injury and Rehabilitation, Beijing, China; ^6^ School of Rehabilitation, Capital Medical University, Beijing, China

**Keywords:** upper urinary tract dysfunction, upper urinary tract urodynamics, ureteral obstruction, mechanosensitive channel, Piezo1

## Abstract

**Objects:** This study investigated changes in upper urinary tract urodynamics (UUTU) after upper urinary tract dysfunction (UUTD).

**Methods:** The UUTD model was induced through unilateral ureteral obstruction. To measure the renal pelvis volume, and resting pressure. Ureteral electromyography (EMG) and *in situ* ureteral constriction experiments were performed. Ureteral tissue was obtained for HE and masson staining, IF staining and IHC research to explore the distribution of Piezo1, and the expression of Piezo1 was studied using Western blotting.

**Results:** The study showed that the renal pelvis volumes and the renal pelvis resting pressures gradually increased post surgery in the experimental group. The degree of ureteral tissue edema, cell necrosis and fibrosis gradually increased. The maximum contraction force and frequency of ureter in the experimental group post surgery were significantly higher than in the sham group. Western blotting showed that the expression intensity of Piezo1 gradually increased and was significantly higher than in the sham group. Further analysis of each sub-layer of the ureter revealed that Piezo1 was highly expressed in the urothelium layer, followed by the suburothelium layer, and had low expression in the smooth muscle cell layer.

**Conclusion:** The study observed that morphological and electrophysiological changes in the upper urinary tract may be important mechanisms of abnormal UUTU. Increased expression of the Piezo1 may be a new molecular mechanism of abnormal urodynamics after UUTD.

## 1 Introduction

The primary function of the upper urinary tract is to deliver urine from the kidneys to the bladder. Upper urinary tract diseases can cause UUTD. Ultimately, abnormal urodynamics of the upper urinary tract may occur, including changes in upper urinary tract neurophysiology, pacing function, and urinary transport dysfunction (Houat et al., 2021). The most common cause of UUTD is ureteral obstruction such as from stones, infections, tumors, lumen compression, and iatrogenic injury (Reicherz et al., 2023). Acute ureteral obstruction cannot only cause clinical symptoms, but can also increase the contraction activity of the upper urinary tract, strengthen the peristalsis of the ureter, and increase intraluminal pressure. Hammad, who studied a rat model of complete unilateral ureteral obstruction, found that the peristaltic frequency of the proximal ureter of the obstruction increased significantly but then gradually decreased. After 2 weeks, the frequency of peristalsis was significantly lower than before obstruction. In the obstructed distal ureter, ureterograde electrical activity gradually disappears, electrical activity was completely undetectable in most regions (Hammad, 2015). Nouaille, who studied electrical activity in a porcine model of chronic ureteral obstruction, found that not all contractions originating in the proximal renal pelvis propagate to the ureter and that obstruction causes a loss of coordination in the frequency of contractions in different parts of the upper urinary tract, which may be due to electrical activity induced through contraction-propagation separation mechanisms (Nouaille et al., 2021). If ureteral obstruction persists, renal interstitial edema, fibroblast proliferation, collagen aggregation, and interstitial fibrosis occurs, leading to decreased glomerular filtration function and renal insufficiency (Oyaert et al., 2020). Therefore, it is particularly important to study the urodynamic abnormal changes and mechanisms after UUTD.

Mechanosensitive ion channels are ubiquitously present in the urinary system and produce a wide range of biologic effects by sensing the mechanical stimuli of shear stress, epithelial cell extension, and fluid (Bagriantsev et al., 2014). Its function is to sense mechanical force changes on the cell membrane, converting the sensed mechanical signals into electrical or chemical signals. Piezo1 is mainly present in non-sensory tissues affected by fluid pressure and flow, such as the cardiovascular, respiratory, digestive, and urinary systems (Wu et al., 2017). Studies have identified many mechanosensitive cells in the digestive system, such as enterochromaffin cells, enteric neurons, smooth muscle cells, and Cajal interstitial cells.

When urothelial cells are stimulated by pressure, fluid shear force, and other abnormal urodynamic factors, Piezo channels are activated and open to generate Ca^2+^ influx. They convert mechanical signals into electrical or chemical signals, and Ca^2+^ is involved in the ureter, the most important ion channel for smooth muscle contraction. Zeng found that when vascular epithelial cells are mechanically stimulated, the depolarization of Piezo1 channel is enhanced, and the voltage-gated Ca^2+^ channel in vascular smooth muscle cells is activated, causing vasoconstriction and an increase in blood pressure. In Piezo1-gene knockout mice, the baroreflex sensitivity of blood vessels decreases, and the mean arterial pressure increases significantly (Zeng et al., 2018).

Using animal experiments expressing Piezo1-fused fluorescent proteins, we found that Piezo1 is expressed in the upper and lower urinary tracts, as well as in the prostate, seminal vesicles, vas deferens, and vagina (Li et al., 2022). Another study found that Piezo1 is also expressed in glomerular endothelial cells, parietal cells, and tubular epithelial cells (Verschuren et al., 2020). Michishita found that the expression of Pieoz1 is increased in the detrusor layer and submucosa, while the nerve fiber NF-L was significantly reduced in rats with partial bladder outlet obstruction. This may be related to the symptoms of urinary storage in patients with benign prostatic hyperplasia. Piezo1 may play a role in compensation related to bladder denervation (Michishita et al., 2016). There is a large amount of Piezo1 in the bladder tissue and a large amount of Piezo2 in the dorsal root ganglion innervating the bladder, which has a clear effect on bladder sensation (Gailly and Devuyst, 2021). After knocking out the Piezo2 gene in dorsal root ganglion neurons, mice lost sensitivity to light touch (Ranade et al., 2014).

So far, the mechanism of the physiologic function of Piezo1 in the urinary tract still needs further study. We believe that Piezo1 may act as a mechanosensitive sensor in the urinary tract and perception of mechanical tension, especially in UUTD, in which the urodynamics are disturbed, resulting in urinary hydrodynamics that lead to changes in Piezo1 in upper urinary tract tissue and then participates in the abnormal mechanisms of UUTU. Therefore, we established an UUTD model to conduct a preliminary study on the UUTU changes and mechanisms.

## 2 Materials and methods

### 2.1 Animal preparation

Ninety 8-week-old female Sprague-Dawley rats weighing 200–220 g (Beijing SPF Biotechnology Co., Ltd., China) were kept in an environment of 22°C, 50%–60% humidity, good ventilation, a 12-h light/dark cycle, and free access to food and water. This study was approved by the Animal Ethics Committee of the China Rehabilitation Research Center (Beijing, China, ID: AEEI-2022-150). All methods are reported in accordance with the ARRIVE guidelines for the reporting of animal experiments.

### 2.2 Establishment of the rat model

Rats were randomly assigned to experimental or sham operation groups. The experimental group was divided into groups at 1, 2, 3, and 4 weeks post surgery, with ten rats per group. Rats were anesthetized using an intraperitoneal injection of 3% sodium pentobarbital. The unilateral ureteral complete obstruction model was established by ligating with a 4-0 silk suture. In the sham group, the ureter was dissociated without ligation.

### 2.3 Measurement of renal pelvis volume

The rats were given limited water 24 h before examination and were anesthetized using intraperitoneal injection of 3% pentobarbital sodium. A Siemens color Doppler ultrasound measured the anterior and posterior diameter (APD), long diameter (L), and transverse diameter (T) of the renal pelvis. Renal pelvis volume was calculated according to the formula: V = APD*L*T*π/6.

### 2.4 Measurement of renal pelvic resting pressure

The rats were given limited water 24 h before examination, when they were anesthetized using an intraperitoneal injection of 3% sodium pentobarbital. The affected kidney was exposed retroperitoneally, and a G18 venipuncture needle was inserted along the back of the kidney into the renal pelvis. A transurethral catheter was placed into the bladder. An MP150 multichannel physiologic recorder (Biopac, United States) was used. The tube of the manometric system was filled with 0.9% saline, the air was expelled, the distal end of the tube was placed in the atmosphere, and zero was set at the same level as the renal pelvis and puncture needle. Then the renal pelvic piezometric tube was connected with a pressure transducer to record the renal pelvic resting pressure smoothly for three times.

### 2.5 *In vivo* ureteral EMG

The rats were anesthetized using inhalation of isoflurane, and the affected side of the ureter was disconnected from the abdomen. EMG was collected at the proximal end of the ureteral obstruction. Bipolar silver needle electrodes (30-μm diameter, NS-S81015-A-24RM, Hailangi, Beijing, China) were inserted into the smooth muscle layer of the ureter. The electrodes were connected to an MP150 multichannel physiologic recorder (Biopac, United States) with high frequency filtering set at 3 kHz and a signal acquisition frequency of 10 kHz. The EMG was analyzed using MP150 software to obtain the area and frequency, which represent the intensity of electrical activity.

### 2.6 *In situ* ureteral constriction experiment

The rats were anesthetized using continuous inhalation of isoflurane, and the ureter was dissociated from the lower abdomen. The distal ureter was ligated with 6-0 silk thread and connected to the suspension of the transducer module, and the electrode was close to the ureteral wall. Complete muscle test systems (Aurora Scientific, 1300A, United States) were used. The optimal electrical stimulation value and optimal stimulation length were obtained according to the software test. When the ureter spontaneous contraction was stable, the maximum contraction intensity of the ureter was obtained using electrical stimulation of 0.8 A. The next stimulation was performed after an interval of 1 min, and the samples in each group were tested for 10 min.

### 2.7 HE staining

The ureteral tissue was fixed in 4% paraformaldehyde and dehydrated with alcohol solution. After dehydration, paraffin sections were made. Paraffin sections were dehydrated again with xylene solution and gradient concentration ethanol solution, cleared and rinsed with distilled water. HE staining were performed in turn. After sufficient staining, the slides were dehydrated and sealed again with gradient alcohol solution. Tissues were visualized using a Leica microscope (Leica, Germany).

### 2.8 Masson staining

Paraffin sections were dehydrated with xylene solution and gradient ethanol solution and rinsed with distilled water. After staining with Weigert iron hematoxylin staining solution, Masson blue solution returned to blue, Ponceau magenta staining solution was stained, and after rinsing with phosphomolybdic acid solution, aniline blue staining solution was stained. After being dehydrated and transparent with xylene solution and gradient ethanol solution, the slices were sealed with neutral gum. Tissues were visualized using a Leica microscope (Leica, Germany). Image-Pro Plus 6.0 software was used to select the same blue color as the unified standard for determining collagen fibers in all photos, and the collagen volume fraction (CVF) (%) was calculated.

### 2.9 Western blotting

The ureter tissues were homogenized in a lysis buffer containing a protease inhibitor cocktail. The protein samples were loaded for each lane and separated by a 6% SDS-PAGE gel. After transferring the blots to a PVDF membrane, they were incubated overnight at 4°C with a rabbit poly-clonal antibody against Piezo1 (1:250, Proteintech, United States) and a rat monoclonal antibody against β-actin (1:1000, Abcam, United Kingdom). These blots were incubated further with a HRP-conjugated secondary antibody and developed in an ECL solution. The optical density (OD) of the strip was analyzed using Image-Pro Plus 6.0 software, and β-actin protein was used as the internal reference. The relative expression of protein was expressed using protein OD/β-actin OD × 100%.

### 2.10 Immunohistochemistry

The ureter tissues were fixed with 4% paraformaldehyde. Then, the ureter tissues were embedded in paraffin, cut into sections, and prepared for immunohistochemistry. After quenching endogenous peroxidase with 3% H_2_O_2_, the tissue slides were incubated with 1% normal rabbit serum to minimize nonspecific binding. Next, the slides were incubated with rabbit polyclonal anti-Piezo1 antibody (1:200, Proteintech, United States) at 37°C for 1 h in a dark, humidified box. After washing with PBS, the slides were incubated with FITC-conjugated goat anti-rabbit IgG (1:100, Abcam, United Kingdom) for 30 min. After washing with PBS, the slides were then incubated with 100 ng/mL DAPI for 10 min. After washing with PBS, the slides were embedded in 50% glycerol. Tissues were visualized using a Leica microscope (Leica, Germany). Immunohistochemical staining mean optical density (MOD) values to quantify staining results. Representative images were obtained with a digital camera and then processed with Image-Pro Plus 6.0 software.

### 2.11 Immunofluorescence staining

The ureter tissues were fixed with 4% paraformaldehyde, then dehydrated with sucrose gradient. Frozen sections were prepared and rinsed with PBS for 10 min. Goat blocking serum was used at 37°C for 30 min, and primary rabbit anti-Piezo1 antibody (1:100, Proteintech, United States) was dropped and incubated overnight in a 4°C refrigerator. The next day, the samples were removed and washed with PBS, and secondary goat anti-rabbit IgG serum (1:300, Abcam, United Kingdom) was added. The samples were incubated at 37°C in the dark for 3 h. After washing with PBS, DAPI solution was added in the dark, and the samples were incubated at 37°C for 30 min. The slides were washed with PBS and mixed in a 1:1 ratio with glycerol and distilled water, sealed, and observed under a fluorescence microscope. Semiquantitative fluorescence intensity analysis was performed using Image-Pro Plus 6.0 software.

### 2.12 Statistical analysis

Statistical analysis was performed using SPSS 26 software. All statistical graphs were drawn using GraphPad Prism 9.0 software. All measurement data were recorded as mean ± standard deviation (X̄ ± SD). We compared 1w, 2w, 3w and 4w in the experimental group with the sham group. We first conducted a normality test on the data, which met the normality test, and carried out one-way ANOVA, which did not meet the normal distribution, and adopted the rank sum test. For normally distributed samples with homogeneity of variance, correlation analysis was performed using Pearson correlation analysis, Spearman correlation analysis was performed on the non-normal distribution data. *p* < 0.05 was considered statistically significant.

## 3 Results

### 3.1 Renal pelvis volume

**FIGURE 1:**
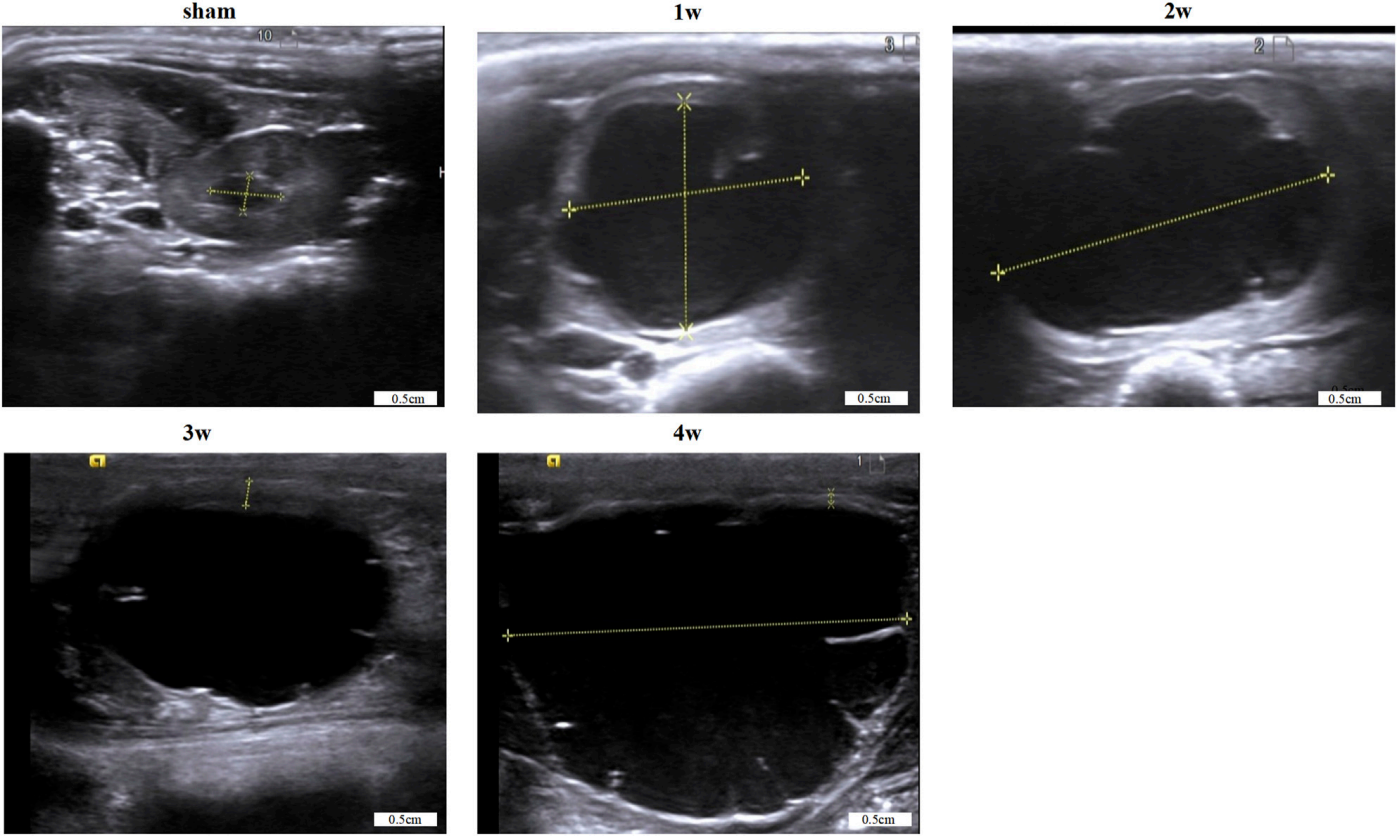
Images of renal pelvis in each group were obtained by B-ultrasound. Scale bars, 0.5 cm.

In the experimental group, the renal pelvis was gradually dilated, the renal parenchyma became thinner, and the hydronephrosis worsened post-surgery. Renal pelvis volume was 1.66 ± 0.14, 2.25 ± 0.15, 2.53 ± 0.10, and 2.97 ± 0.14 cm^3^ at 1, 2, 3, and 4 weeks, respectively, significantly higher than 0.10 ± 0.02 cm^3^ in the sham group (*p* < 0.0001). (Table 1; Figure 1).

**TABLE 1:** Renal pelvis volume (X̄ ± SD): cm^3^.

Sham	1w	2w	3w	4w
0.10 ± 0.02	1.66 ± 0.14****	2.25 ± 0.15****	2.53 ± 0.10****	2.97 ± 0.14****

Note: ****: Compared with sham group: *p* < 0.0001.

### 3.2 Renal pelvis resting pressure

The renal pelvis resting pressure of the experimental group was 11.41 ± 0.64, 12.30 ± 0.67, 12.96 ± 1.76, and 13.70 ± 1.32 cm H_2_O at 1, 2, 3, and 4 weeks post-surgery, respectively, higher than that of the sham group: 8.29 ± 0.98 cm H_2_O. Compared 1w with sham group (*p* < 0.01); Compared 2w, 3w, 4w with sham group (*p* < 0.0001) (Table 2).

**TABLE 2:** Renal pelvis resting pressure (X̄ ± SD): cmH_2_O.

Sham	1w	2w	3w	4w
8.29 ± 0.98	11.41 ± 0.64**	12.30 ± 0.67****	12.96 ± 1.76****	13.70 ± 1.32****

Note: Compared 1w with sham group: **p < 0.01; Compared 2w, 3w, 4w with sham group: ****p < 0.0001.

### 3.3 *In vivo* ureteral EMG

**FIGURE 2:**
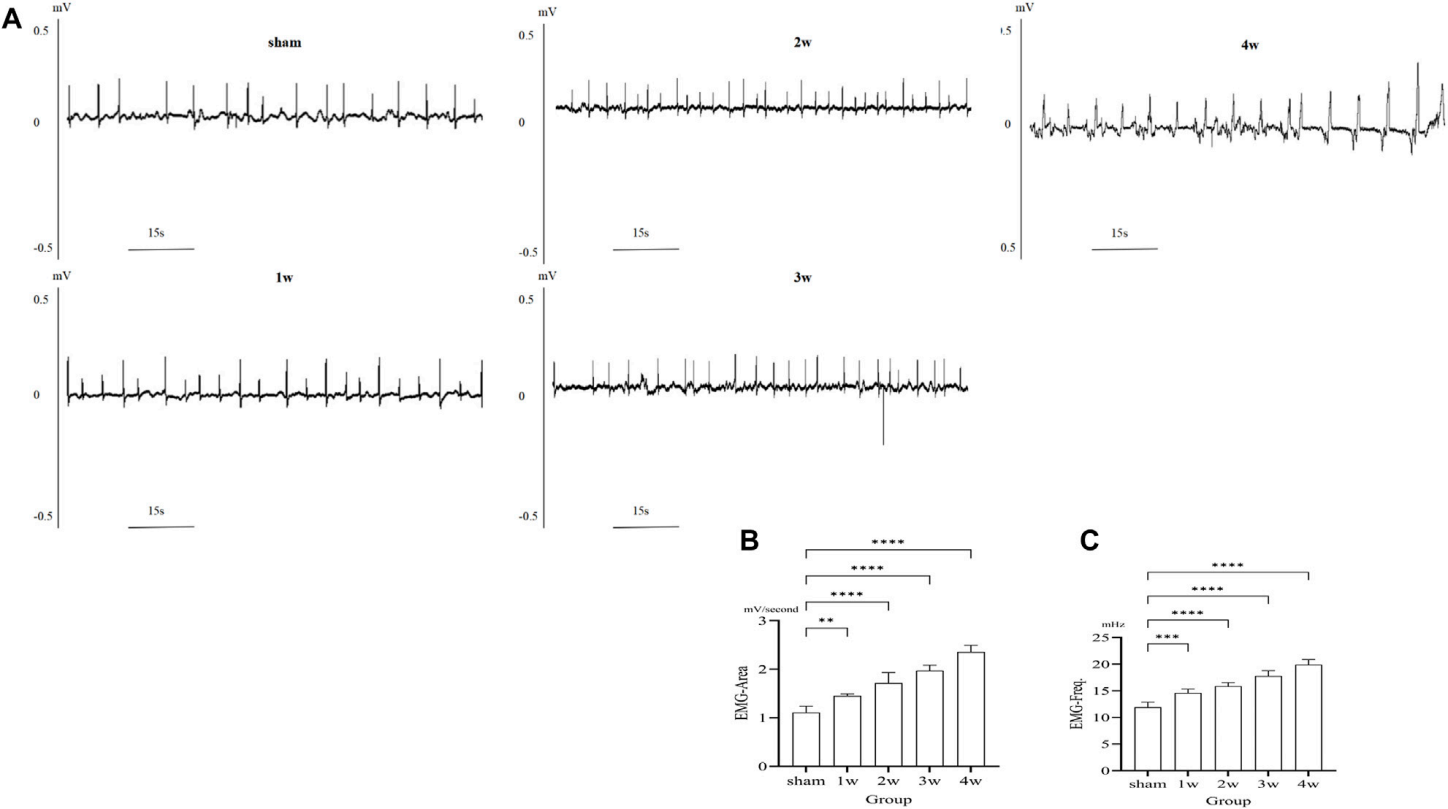
**(A)** EMG images in different groups. **(B)** The analysis the EMG-area shows compared the 1 week with sham group: *p* < 0.01, compared the 2, 3, 4 weeks respectively with sham group: *p* < 0.0001. **(C)** The analysis the EMG-frequency shows compared the 1week with sham group: *p* < 0.001, compared the 2, 3, 4 weeks respectively with sham group: *p* < 0.0001. **p < 0.01, ***p < 0.001, ****p < 0.0001.

After ureteral obstruction, the intensity of electrical activity in the ureter is significantly enhanced and changes with time. The intensity of ureteral electrical activity increased gradually compared with the sham group (Figure 2A). EMG-area analysis of ureteral EMG found that at 1 week post-surgery is 1.33 ± 0.04 mV/s, stronger than that of the sham group 1.11 ± 0.13 mV/s (*p* < 0.01); EMG-area at 2, 3, 4 weeks post-surgery are 1.71 ± 0.22, 1.97 ± 0.11, and 2.36 ± 0.14 mV/s, significantly stronger than the sham group 1.11 ± 0.13 mV/s (*p* < 0.0001) (Figure 2B). EMG-frequency analysis found that at 1 week post-surgery is 14.58 ± 0.73 mHz, higher than that of the sham group 11.91 ± 0.95 mHz (*p* < 0.001) and at 2, 3, 4 weeks post-surgery are 15.86 ± 0.66, 17.76 ± 1.03, and 19.90 ± 0.96 mHz, significantly higher than the sham group 11.91 ± 0.95 mHz (*p* < 0.0001) (Figure 2C).

### 3.4 *In situ* ureteral contraction experiment

**FIGURE 3:**
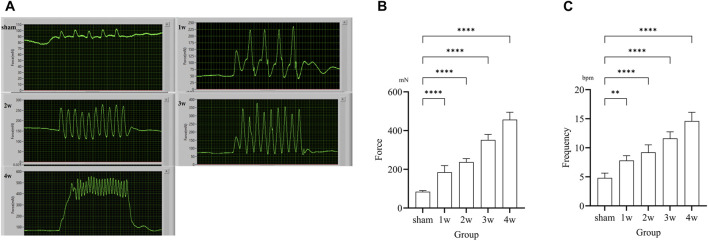
**(A)** Images of *in situ* ureteral contraction experiment in different groups. **(B)** The analysis the maximum contraction shows compared the 1, 2, 3, 4 weeks with sham group: *p* < 0.0001. **(C)** The analysis of frequency shows compared the 1 week with sham group: *p* < 0.01, compared the 2, 3, 4 weeks respectively with sham group: *p* < 0.0001. **p < 0.01, ****p < 0.0001.

Following ureteral obstruction, the strength and frequency of contractile force in the ureter were significantly higher than in the sham group and significantly enhanced and changes with time (Figure 3A). The maximum contraction force of the ureter at 1, 2, 3, and 4 weeks post-surgery were 184.65 ± 34.38, 291.06 ± 44.55, 391.13 ± 46.47, and 476.70 ± 44.95 mN, significantly stronger than those in the sham group: 84.02 ± 6.18 mN (*p* < 0.0001) (Figure 3B). The contraction frequency at 1 week is 7.6 ± 0.89, higher than the sham group: 4.8 ± 0.84 bpm (*p* < 0.01); And at 2, 3, 4 weeks are 9.2 ± 1.30, 11.6 ± 1.14, and 14.6 ± 1.52 bpm, respectively, significantly higher than sham group: 4.8 ± 0.84 bpm (*p* < 0.0001) (Figure 3C).

### 3.5 HE staining

**FIGURE 4:**
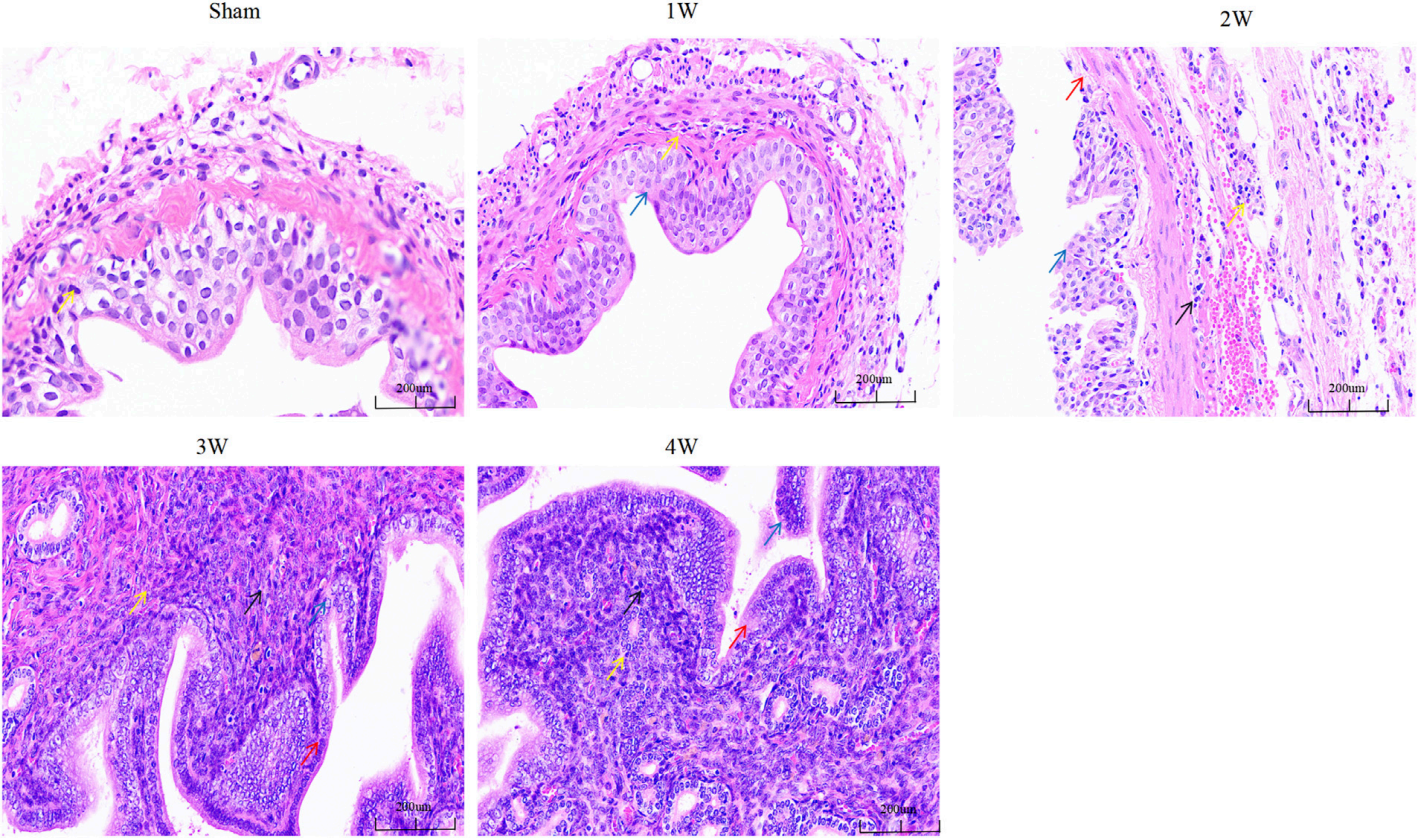
The ureteral tissue HE staining in each group. Note: Yellow arrow: inflammatory cells; Blue arrow: edematous epithelial cells; Red arrows: exfoliated epithelial cells; Black arrow: necrotic cells. Scale bars, 200 µm.

The degree of structural abnormality of the ureter was gradually aggravated, and inflammatory cell infiltration was observed in the tissue. The degree of local mucosal edema in the visual field was gradually aggravated, the cells were swollen, and the cytoplasm was lightly stained. At 2, 3, and 4w, the epithelial cells shed and the lamina propria was exposed. The degree of cell necrosis was significantly aggravated, the nuclei were fragmented, and the pyknosis was deeply stained (Figure 4).

### 3.6 Masson staining

**FIGURE 5:**
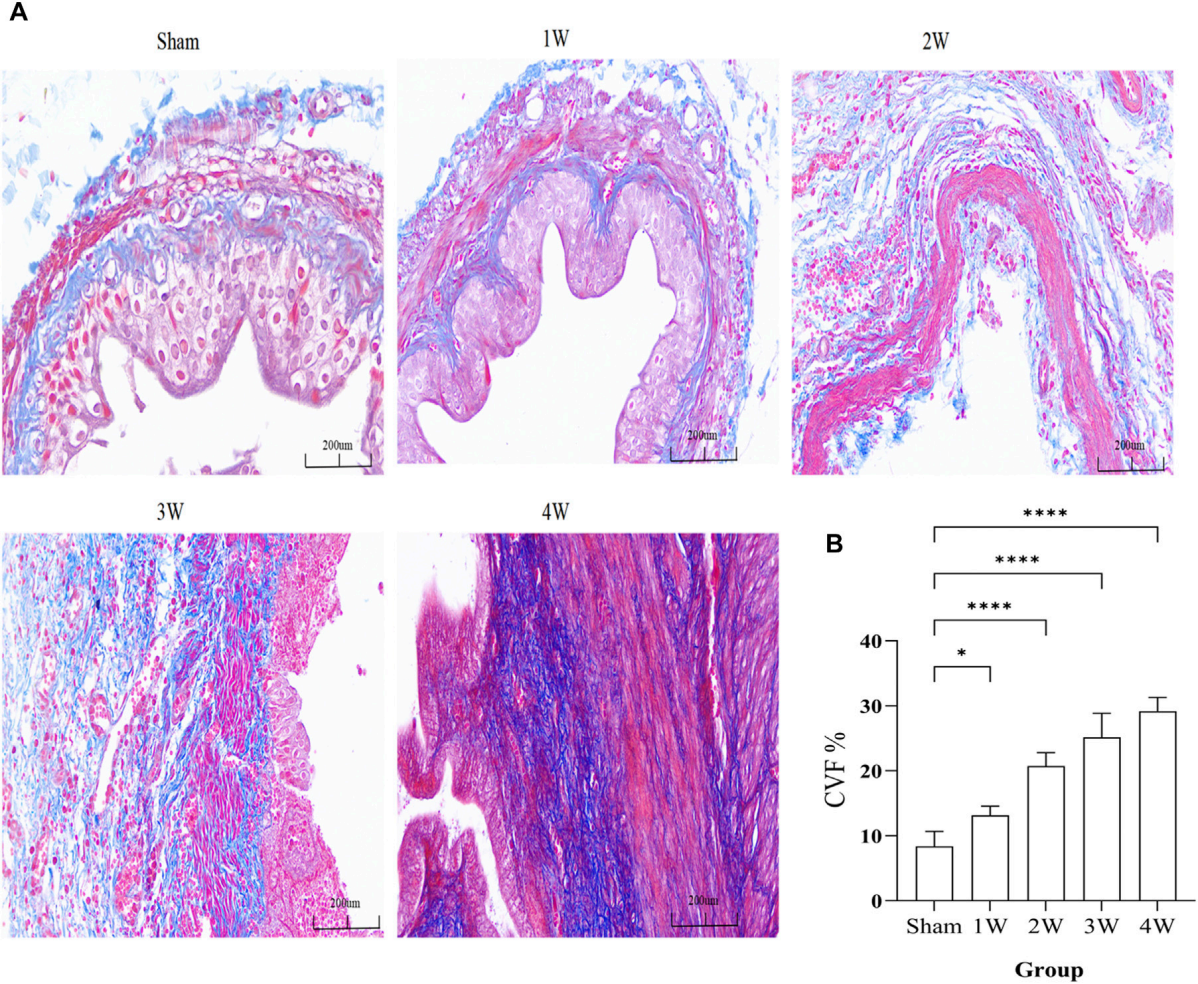
**(A)** The ureteral tissue masson staining in each group. **(B)** The analysis the CVF in different groups. Compared the 1 week with sham group: *p* < 0.05, compared the 2, 3, 4 weeks respectively with sham group: *p* < 0.0001. Scale bars, 200 µm *p < 0.05, ****p < 0.0001.

The fibrosis of ureteral tissue gradually increased, and CVF was significantly higher than that of sham group (Figure 5A). Compared the CVF at 1 week with sham group (*p* < 0.05), compared the CVF at 2, 3, 4 weeks respectively with sham group the difference was significant (*p* < 0.0001) (Figure 5B).

### 3.7 Western blotting and correlation analysis

**FIGURE 6:**
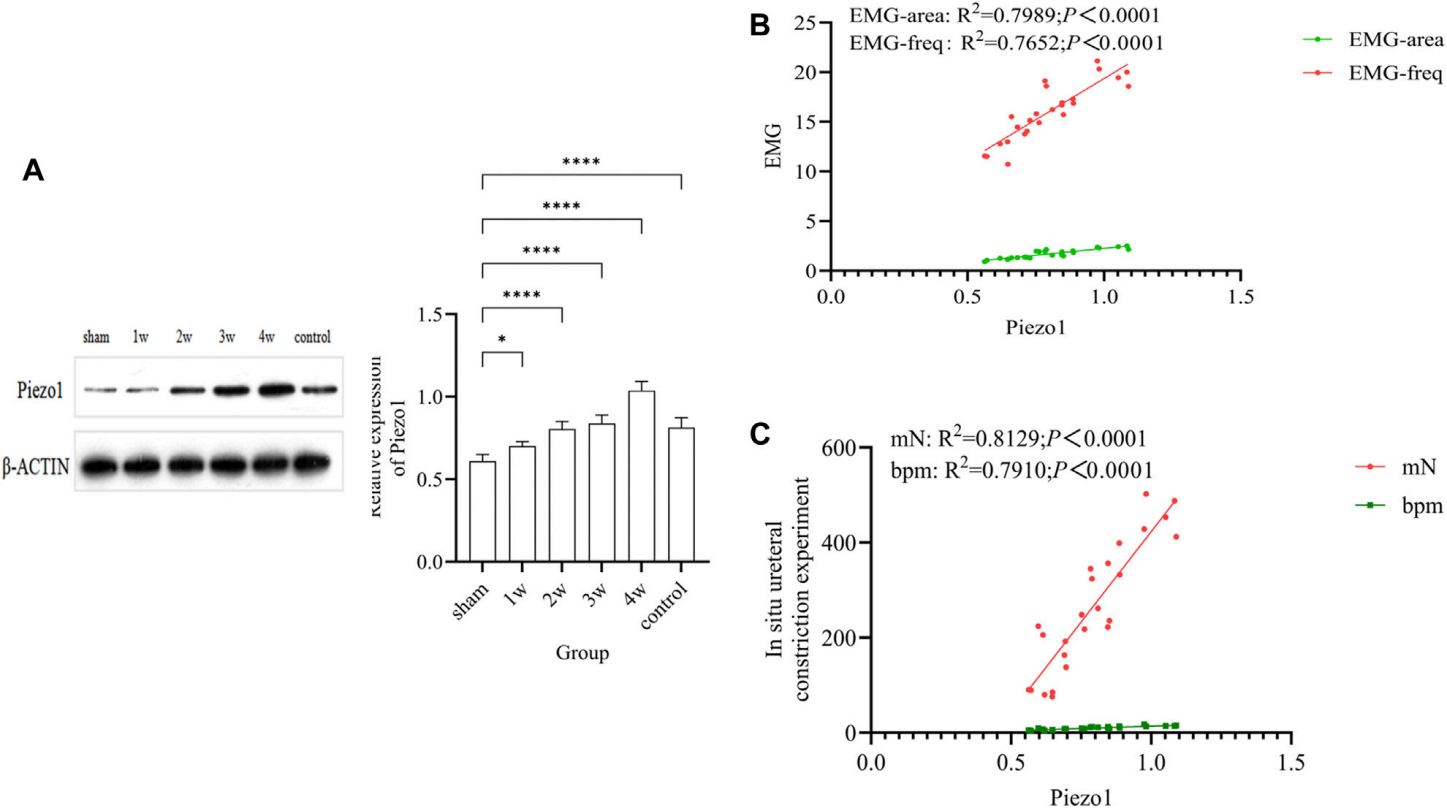
**(A)** The expression of Piezo1 in each group and quantitative analysis: compared 1w with sham group (*p* < 0.05); compared 2w, 3w, 4w, control group respectively with sham group (*p* < 0.0001). **(B)** Analysis of the correlation between quantitative Piezo1 expression and the strength of ureteral EMG activity: EMG-area: R^2^ = 0.7989, *p* < 0.0001; EMG-freq: R^2^ = 0.7652, *p* < 0.0001. **(C)** Analysis of the correlation between quantitative Piezo1 expression and the strength of ureteral ureteral contractility: mN: R^2^ = 0.8129, *p* < 0.0001; bpm: R^2^ = 0.7910, *p* < 0.0001. *p < 0.05; ****p < 0.0001.

The expression intensity of Piezo1 in the experimental group was gradually increased, and the quantitative analysis were: compared 1w with sham group (*p* < 0.05); compared 2w, 3w, 4w, control group respectively with sham group (*p* < 0.0001) (Figure 6A). Quantitative Piezo1 expression was positively correlated with the strength of EMG activity in the ureter (EMG-area: R^2^ = 0.7989, *p* < 0.0001; EMG-freq: R^2^ = 0.7652, *p* < 0.0001) (Figure 6B) and with the strength of ureteral contractility (mN: R^2^ = 0.8129, *p* < 0.0001; bpm: R^2^ = 0.7910, *p* < 0.0001) (Figure 6C).

### 3.8 Immunohistochemistry

**FIGURE 7:**
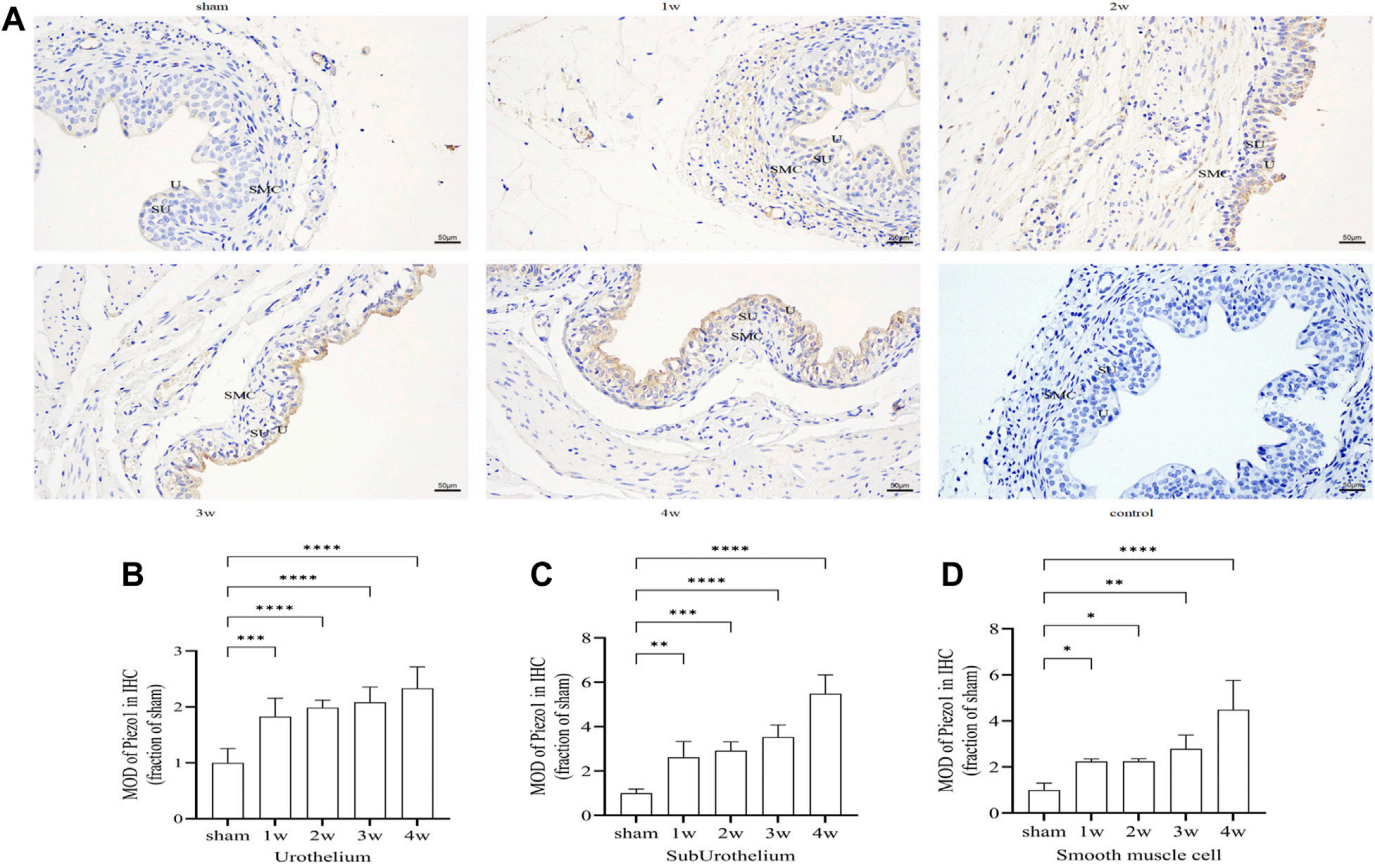
**(A)** Immunohistochemistry expression of Piezo1 in ureteral tissue. Piezo1 was expressed in all layers of the ureter, There was no significant positive expression in the negative control group. **(B)** The expression intensity of Piezo1 in the urothelium layer at 1 week compared with the sham group (*p* < 0.001); at 2, 3, 4 weeks post-surgery compared with the sham group (*p* < 0.0001). **(C)** In the suburothelium layer compared the sham group with 1 week (*p* < 0.01), with 2 weeks (*p* < 0.001), at 3, 4 weeks compared with the sham group (*p* < 0.0001). **(D)** In the smooth muscle cell layer, compared 1, 2 weeks respectively with the sham group (*p* < 0.05), compared the sham group with 3 weeks (*p* < 0.01), with 4 weeks (*p* < 0.0001). U, urothelium; SU, sub-urothelium; SMC, smooth muscle cell. *p < 0.05; **p < 0.01; ***p < 0.001; ****p < 0.0001. Scale bars, 50 µm.

Piezo1 was expressed in the ureteral tissue. Further analysis of the expression intensity of Piezo1 in the sub-layers showed that Piezo1 was highly expressed in the urothelium layer, followed by the urothelium and smooth muscle cell layers, There was no significant positive expression in the negative control group (Figure 7A). Quantitative analysis showed that the expression intensity of Piezo1 in the urothelium layer at 1 week compared with the sham group (*p* < 0.001); at 2, 3, 4 weeks post-surgery compared with the sham group (*p* < 0.0001) (Figure 7B). In the suburothelium layer compared the sham group with 1 week (*p* < 0.01), with 2 weeks (*p* < 0.001), at 3, 4 weeks post-surgery compared with the sham group (*p* < 0.0001) (Figure 7C). In the smooth muscle cell layer, expression intensity compared 1, 2 weeks respectively with the sham group (*p* < 0.05), compared the sham group with 3 weeks (*p* < 0.01), with 4 weeks (*p* < 0.0001) (Figure 7D).

### 3.9 Immunofluorescence staining

**FIGURE 8:**
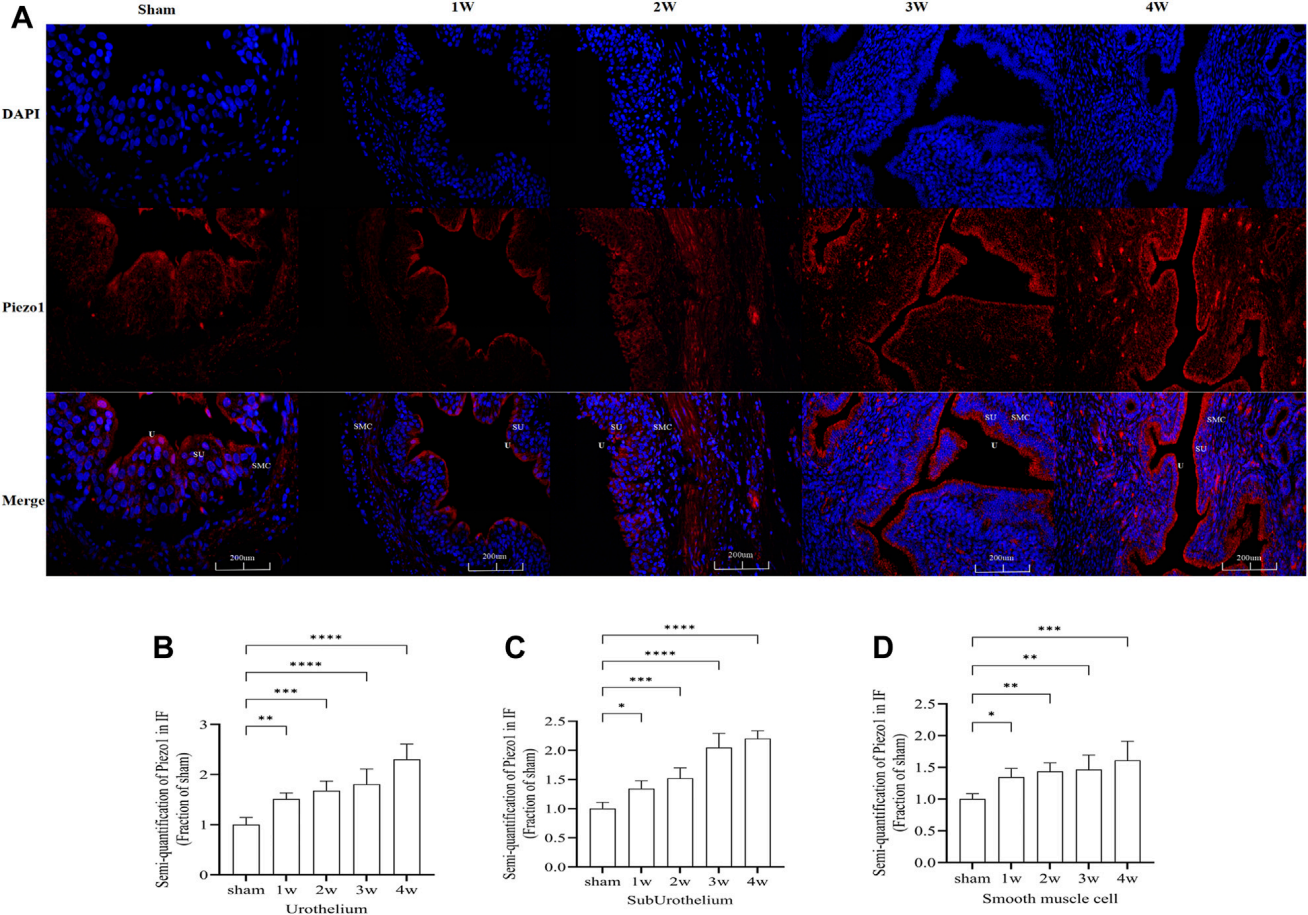
**(A)** Immunofluorescence expression of Piezo1 in ureteral tissue, was found in all layers of the ureter. Red fluorescence indicates target protein expressing cells. **(B)** Compared the Piezo1 expression intensity in the urothelium layer at sham group with 1 week: *p* < 0.01), with 2 weeks: *p* < 0.001, with 3 and 4 weeks: *p* < 0.0001. **(C)** Compared the Piezo1 expression intensity in the suburothelium layer at sham group with 1 week: *p* < 0.05, with 2 weeks: *p* < 0.001, with 3 and 4 weeks: *p* < 0.0001. **(D)** Compared the Piezo1 expression intensity in the smooth muscle cell layer at sham group with 1 week: *p* < 0.05, with 2 and 3 weeks: *p* < 0.01, with 4 weeks: *p* < 0.001. U, urothelium; SU, suburothelium; SMC, smooth muscle cell. *p < 0.05; **p < 0.01; ***p < 0.001; ****p < 0.0001. Scale bars: 200 µm.

Piezo1 was distributed in the ureteral tissue. Further analysis of the sublayers of the ureter showed that Piezo1 was highly expressed in the urothelium layer, followed by the suburothelium and smooth muscle cell layers (Figure 8A). Semi-quantitative analysis of Piezo1 expression intensity found that the intensity in the urothelium layer compared the sham group with 1 week (*p* < 0.01), with 2 weeks (*p* < 0.001), with 3 and 4 weeks (*p* < 0.0001) (Figure 8B). The expression intensity in the suburothelium layer compared the sham group with 1 week (*p* < 0.05), with 2 weeks (*p* < 0.001), with 3 and 4 weeks (*p* < 0.0001) (Figure 8C). The expression intensity in the smooth muscle cell layers compared the sham group with 1 week (*p* < 0.05), with 2 and 3 weeks (*p* < 0.01), with 4 weeks (*p* < 0.001) (Figure 8D). This indicates that Piezo1 expression changes in the urothelium and suburothelium layers, which are significantly higher than in the smooth muscle cell layer.

## 4 Discussion

UUTD refers to abnormal urodynamics of the upper urinary tract that interferes with its physiologic ability to deliver urine and can lead to renal insufficiency or even renal failure. The common causes of UUTD include disorders such as upper urinary tract obstruction, neurogenic injury, and lower urinary tract dysfunction. Ureteral obstruction caused by stones, trauma, infection, and iatrogenic ureteral injury is very common in clinical practice. In recent years, increasing attention has been paid to the study of UUTU, which cannot only assist in diagnosis of upper urinary tract diseases but also provide evidential support for treatment and follow-up. Our study systematically investigated UUTU in terms of morphology, electrophysiologic function, and mechanosensitive pathways involved in upper urinary tract peristalsis by establishing a ureteral obstruction model.

One of the keys to UUTU research is measurement of the upper urinary tract pressure. Under normal physiologic conditions, increased pressure facilitates urine delivery, but excessive pressure leads to abnormal urodynamics. The study of intraluminal pressure can help determine the relationship between obstruction and clinical symptoms. Whitaker (Whitaker, 1973) was the first to establish percutaneous renal pelvic perfusion manometry, which can assist in diagnosis of upper urinary tract obstruction and degree of obstruction by measuring renal pelvic pressure. Our study found that renal pelvis volume was 1.66 ± 0.14, 2.25 ± 0.15, 2.53 ± 0.10, and 2.97 ± 0.14 cm^3^ at 1, 2, 3, and 4 weeks, respectively, after ureteral obstruction, significantly higher than that of the sham group: 0.10 ± 0.02 cm^3^. The renal pelvic resting pressure of the experimental group was 11.41 ± 0.64, 12.30 ± 0.67, 12.96 ± 1.76, and 13.70 ± 1.32 cm H_2_O 1, 2, 3, and 4 weeks post-surgery, higher than that of the sham group: 8.29 ± 0.98 cm H_2_O.

Increased renal pelvic pressure leads to increased retrotubular pressure, which may cause glomerular atrophy, basement membrane hyperplasia, renal tubular dilatation, and interstitial fibrosis and affect the glomerular filtration rate (Calle et al., 2021). Animal studies have found that even though the glomerular filtration rate returns to baseline when the ureter is relieved 24–72 h after unilateral ureteral obstruction, longitudinal studies have found impaired urinary concentration, albumin leakage, interstitial fibrosis, altered pro-inflammatory and pro-apoptotic markers, and impaired response of the obstructed kidney to stimuli (Hammad et al., 2014; Hu et al., 2019). Our study also found that the degree of edema and fibrosis of ureteral epithelial cells gradually increased after ureteral obstruction, and epithelial cell exfoliation and interstitial cell necrosis may occur in the later stage. The pressure, stress, deformation, and shear stress of the ureteral lumen change after obstruction also must be considered. The anatomic structure after obstruction, potential energy, and the pressure and flow rate of urine may affect urine transport. A fluid-structure coupling analysis using three-dimensional reconstruction and simulation model of ureteral and urethral obstruction, showed that increasing urinary stricture may gradually increase the shear stress on the wall of the urinary tract and the pressure at the entrance of the stricture and accelerate the urine flow rate (Ishii et al., 2015; Shilo et al., 2020). These factors may be the hydrodynamic mechanisms leading to abnormal urodynamics.

Urine transport in the upper urinary tract is driven by the contraction of ureteral smooth muscle. Smooth muscle cells depolarize by reaching the threshold of a depolarized action potential, causing smooth muscle excitatory contraction. Lang et al. (2002) identified sympathetic and parasympathetic nerve receptors in the smooth muscle of the upper urinary tract, and norepinephrine and acetylcholine can change the activity of smooth muscle. Under physiologic conditions, urine is transported in the form of a urine bolus in the upper urinary tract. Thus, ureteral urination is intermittent, powerful, and jetted. The rate of ureteral contraction in healthy people is 3 or 4 times per minute. The interval between two peristalsis contractions was 7–9 s, and the speed of peristalsis was 2–6 cm per second (Griffiths, 1987).

The study of the nerve electrical activity and muscle tension of ureteral smooth muscle is also an important research topic in UUTU. Our study found that during ureteral obstruction, the electrical activity of ureteral smooth muscle was significantly increased by *in vivo* EMG and *in situ* ureteral contraction experiment. The area under the EMG curve and the frequency of EMG at 1, 2, 3, and 4 weeks after obstruction were higher than the sham group. The maximum contraction force and frequency of ureters at 1, 2, 3, and 4 weeks post-surgery were significantly higher than in the sham group: 84.02 ± 6.18 mN and 4.8 ± 0.84 bpm, respectively.

Although we did not study changes in the related neuroreceptors in the ureteral smooth muscle, we found that the abnormal UUTU after obstruction enhanced the electrical activity and tone of the smooth muscle to overcome the effect of obstruction on urine delivery. The peristaltic and contractile functions of the ureter may be affected at any part of the upper urinary tract after obstruction. However, due to the limitations of detection methods, it has not been applied to the clinical ureteral neurophysiologic examination to assist the diagnosis of patients with UUTU abnormalities.

Mechanosensitive ion channels convert mechanical signals into electrochemical signals, participate in signal transduction between cells, maintain the normal physiologic functions of the body, and are essential components of almost all mammals. The urinary system is an important metabolic pathway that regulates the balance of water, salt, and electrolytes and plays an important role in maintaining the stability of the internal environment in humans. As a versatile mechanosensitive sensor in mammals, Piezo channels are involved in the regulation of the normal physiologic processes of the urinary system by sensing mechanical stimuli such as shear stress and bladder wall stretch (Xu et al., 2020). Therefore, it is important to investigate the expression and mechanism of Piezo channels in the urinary system. In the animal study, Piezo1 was found to be overexpressed in the kidney, urethra, bladder, and ureter. An immunofluorescence staining study showed that Piezo1 was mainly expressed in glomerular endothelial cells, Bowman’s capsule parietal cells, distal convoluted tubule endothelial cells, and urothelial cells (Martins et al., 2016). In addition, other studies have found that Piezo1 is also expressed in urothelial stromal cells and smooth muscle cells, but mainly in urothelial cells. Piezo1 has also been found in tissues such as the urethra, sphincter cells, vaginal epithelium, prostate, seminal vesicles, and ejaculatory ducts (Miyamoto et al., 2014). Although studies have confirmed that Piezo1 is widely distributed in the urinary system, its functions need further exploration. Studies have found that Piezo expression in human and mouse bladder cancer, and the expression of Piezo1 and Piezo2 was significantly increased in the tissues. In addition, Piezo1 expression may be related to tumor stage, grade, and size (Etem et al., 2018).

In our study, Piezo1 was widely present in the ureteral tissue, consistent with the previously mentioned findings. The quantitative analysis of WB showed that the expression intensity of Piezo1 in experimental groups at 1, 2, 3, and 4 weeks post-surgery increased gradually and was significantly higher than in the sham group. IF staining and IHC showed that Piezo1 was widely present in the urothelium, sub-urothelium, and smooth muscle cell layers of the ureter. The expression intensity of Piezo1 in the ureteral tissue peaked 4 weeks post-surgery. The expression intensity of Piezo1 in the urothelium layer and sub-urothelium were significantly higher than smooth muscle cell layer.

We conclude that increased pressure in the lumen, shear stress in the wall, and inconsistent urine flow at the proximal and distal end of the stricture stimulate urothelial cells, leading to enhanced expression of Piezo1. We also found that the expression of Piezo1 in the urothelium layer and sub-urothelium were significantly higher than in the and muscle cell layers after obstruction. This is because the contraction and peristalsis of the upper urinary tract depend on pacemaker cells in the interstitium of the upper urinary tract. Studies have identified Cajal as the main pacemaker cell; it mainly exists in the suburothelium and urothelium layers of the urinary tract (Lang and Hashitani, 2019). After ureteral obstruction, the peristalsis and contraction rhythm of the ureter change, and the delivery of urine in the ureter is limited. To overcome the abnormal peristalsis mechanism of the upper urinary tract caused by obstruction, the upper urinary tract compensates with the expression of Piezo1, which causes Cajal pacemaker cells to enhance the pacing frequency and intensity and increases the electrical activity and muscle tone of the ureteral smooth muscle, consistent with our previous findings. The epithelial cells also gradually shed after obstruction, while the urothelium interstitial cells and smooth muscle cells proliferate, resulting in the differential expression of Piezo1 in different sublayers.

A study (Zhao et al., 2022) found that the expression of Piezo1 protein significantly increased in the fibrotic kidney after unilateral ureteral obstruction in humans and mice compared with the control group, especially in the proximal renal tubular epithelial cells, which may be activated and upregulated through renal tubular epithelial cells’ response to mechanical stress. However, the early activation and upregulation of Piezo1 after unilateral ureteral obstruction may be attributed to the increased pressure in the lumen of the tract or the mechanical stretch of the epithelial cells caused by the dilatation of the upper urinary tract and the hydrops urine pool (Quinlan et al., 2008). In addition, the use of Piezo1 inhibitor GsMTx4 can significantly improve the degree of renal fibrosis caused by the unilateral ureter (Bae et al., 2011). This strongly suggests that Piezo1 plays an important role in UUTD caused by ureteral obstruction and the development of renal fibrosis.

Our study also has limitations. First, the urodynamic manifestations of UUTD caused by partial or complete ureteral obstruction may not be consistent. We have conducted only a 4 weeks study of complete ureteral obstruction, and follow-up studies are needed. In addition, the expression of Piezo1 in the obstruction group was significantly higher than that in the sham group, but the specific mechanism of Piezo1 involvement still needs to be explored, especially the functional connection with upper urinary tract pacemaker cells (e.g., Cajal cells), which affects abnormal upper urinary tract dynamics.

## 5 Conclusion

After establishing a common model of UUTD following ureteral obstruction, we found that renal pelvis volume and resting pressure significantly increased after obstruction, and neuroelectrophysiological activity and muscle contraction of the upper urinary tract smooth muscle were significantly enhanced. Morphological and electrophysiological changes in the upper urinary tract may be important mechanisms of abnormal UUTU. We also explored Piezo1 expression was positively correlated with the strength of EMG activity in the ureter and with the strength of ureteral contractility after ureteral obstruction, but the specific mechanism still needs further study.

## Data Availability

The raw data supporting the conclusion of this article will be made available by the authors, without undue reservation.
